# A Green-Synthesized Fluorescent Carbon Dot Probe Derived from Banana Peel for Cellular Imaging and Sensing of Tetracycline

**DOI:** 10.3390/ma18225211

**Published:** 2025-11-17

**Authors:** Sihua Zeng, Chunrong Qin, Yuzhu Zhang, Haoyu Chen, Hua Lin

**Affiliations:** 1 Guangxi Key Laboratory of Environmental Pollution Control Theory and Technology, Guilin University of Technology, Guilin 541006, China; 202211002@hzxy.edu.cn (S.Z.); zhangyuzhu1128@163.com (Y.Z.); 2 University Engineering Research Center of Watershed Protection and Green Development, Guilin University of Technology, Guilin 541006, China; 3 Guangxi Key Laboratory of Calcium Carbonate Resources Comprehensive Utilization, College of Materials and Chemical Engineering, Hezhou University, Hezhou 542899, China; 14777562116@163.com (C.Q.); 19197800145@163.com (H.C.)

**Keywords:** banana peel, carbon quantum dots, tetracycline, cell imaging, intracellular detection

## Abstract

The valorization of biomass waste represents an important direction in green chemistry. This study successfully prepared blue fluorescent carbon dots (BP-CDs) from waste banana peels via a one-step hydrothermal method, establishing a dual-functional platform for both pollutant detection and cellular imaging. The resulting material exhibited uniform particle size (~2.05 nm), good water dispersibility, and strong fluorescence emission at 445 nm under 360 nm excitation. It maintained over 93% of its initial fluorescence intensity after 20 days, demonstrating excellent stability. Based on the inner filter effect, the probe enabled a highly selective detection of tetracycline with a detection limit of 0.191 µM and two wide linear ranges (0–15 µM, R^2^ = 0.996; 15–95 µM, R^2^ = 0.991). Cellular experiments confirmed the good biocompatibility of BP-CDs (cell viability > 84%) and their successful application in cell imaging. More importantly, the probe achieved visual observation and semi-quantitative analysis of the distribution and content of tetracycline in living cells, providing a direct tool for studying the cellular behavior of antibiotics. This work not only offers a new strategy for banana peel valorization but also develops a green fluorescence imaging platform suitable for tracking intracellular pollutants.

## 1. Introduction

Banana stands as one of the most prolific fruit crops worldwide, with an annual production surpassing 120 million tons, consequently generating nearly 40 million tons of banana peel waste [1,2]. This substantial byproduct poses a pressing environmental challenge, as it releases potent greenhouse gases like methane or unpleasant odors during decomposition in landfills [3]. To promote the resource utilization of this waste stream, researchers have explored various conversion pathways [4]. Conventional methods typically involve the production of organic fertilizers, compost, or animal feed [5,6]. In the realms of materials science and energy, the abundant cellulose and lignin constituents of banana peels can be harnessed for synthesizing bioplastics or converted into bioethanol via fermentation [7,8]. Furthermore, the peels contain bioactive compounds, including antioxidants, carotenoids, biogenic amines, and polyphenols, which offer potential as raw materials for pharmaceutical and cosmetic applications [9,10]. In water treatment, the naturally porous structure and active functional groups of banana peels have demonstrated remarkable efficacy in adsorbing and removing heavy metal ions, organic dyes, phenols, antibiotics and pesticides [11]. While existing studies validate the feasibility of transforming banana peels into high-value materials, current technological pathways remain inadequate to manage the immense global output. Thus, the development of diverse, efficient functional materials from this waste and the expansion of their cross-disciplinary applications are pivotal for achieving comprehensive and high-value utilization.

Antibiotic pollution has emerged as a global ecological and public health challenge. Among various antibiotics, tetracycline (TC) is a foundational agent for the prevention and treatment of zoonotic diseases, ranking second in global usage [12]. This compound is chemically stable, resistant to degradation, and exhibits significant bioaccumulation potential. Its widespread release not only disrupts ecological balance but can also be enriched through the food chain, ultimately inducing health risks such as hepatotoxicity and abnormal skeletal development [13]. As countries increasingly tighten the maximum residue limits for tetracycline in food, the development of efficient and sensitive detection technologies has become particularly critical [14]. Conventional methods for detecting TC residues, such as capillary electrophoresis [15], Raman spectroscopy [16], immunochromatographic assay [17], chromatography-mass spectrometry [18], and high-performance liquid chromatography [19], offer high accuracy but involve complex procedures and reliance on sophisticated instrumentation, limiting their suitability for rapid on-site testing. In contrast, fluorescence sensing technology has emerged as a highly promising alternative due to its high sensitivity, operational simplicity, and rapid response. However, existing research predominantly focuses on in vitro analysis of animal tissue homogenates or extracts, lacking effective strategies for the in situ visualization and tracking of TC at the living cellular level [20,21]. Achieving in situ detection within cells requires probes that possess exceptional biocompatibility and cell permeability, ensuring they perform their detection function without causing cellular damage and maintain stable performance in complex biological environments. Nevertheless, many reported fluorescent sensing materials, including organic molecule probes [22], nanoparticles [23], metal–organic frameworks (MOFs) [24], and semiconductor quantum dots [25], face a fundamental challenge of inadequate biocompatibility. Their potential cytotoxicity significantly hampers application in biological fields such as cellular imaging. Therefore, the development of a fluorescent probe that combines a green synthesis background, excellent biocompatibility, and suitability for in situ tracking of intracellular TC is of great importance for gaining deeper insights into its cytotoxic mechanisms and fate within organisms.

**Figure 1:**
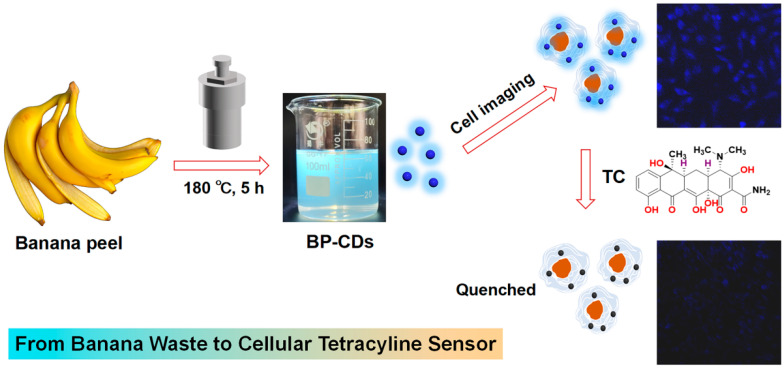
Schematic illustration of converting banana peel waste into fluorescent carbon dots for intracellular detection of TC.

Addressing the dual challenges of biomass waste utilization and the technical limitations in intracellular TC tracking, this study converted fresh banana peels into fluorescent carbon dots (BP-CDs) via a one-pot hydrothermal method (180 °C, 5 h) (Figure 1). The resulting material exhibits small size, excellent water dispersibility, stable fluorescence, and good biocompatibility. A sensing platform constructed based on the inner filter effect demonstrated high sensitivity for TC detection in aqueous environments. The most significant breakthrough lies in our achievement of direct visualization and semi-quantitative analysis of intracellular TC distribution by leveraging these properties. This “waste-to-probe” strategy not only opens a new avenue for the valorization of agricultural waste but also provides a powerful tool for investigating antibiotic behavior at the cellular level.

## 2. Materials and Methods

### 2.1. Materials

Tetracycline hydrochloride (TC), Ceftazidime Sodium (CTR), Chloramphenicol (CAP), Amoxicillin (AMX), Kanamycin sulfate (KAN), Erythromycin (EM), NaOH, NaCl, CuCl_2_, CaCl_2_, ZnCl_2_, CdCl_2_, MnCl_2_, K_2_Cr_2_O_7_, FeCl_3_, Ni(NO_3_)_2_, HCl, CoSO_4_, KCl, and Hg(NO_3_)_2_ of analytical grade were procured from Sinopharm Chemical Reagent Co., Ltd. (Shanghai, China) and used as received. 3-(4,5-Dimethylthiazol-2-yl)-2,5-diphenyltetrazolium bromide (MTT, analytical grade) was sourced from Innochem Co. (Shanghai, China). Mouse melanoma cells (B16) and fibroblast (L929) cells were obtained from the Stem Cell Bank of the Chinese Academy of Sciences (Shanghai, China). Fresh banana peels were collected from a local market in Guilin, China. Ultrapure water (18.25 MΩ·cm) was used throughout all experiments.

### 2.2. Characterization

The physicochemical properties were systematically analyzed as follows: morphology was observed by Transmission Electron Microscope (TEM) (JEOL JEM-F200, Tokyo, Japan). Chemical structure was characterized by Fourier Transform infrared spectroscopy (FTIR) (Nicolet Nexus 470, KBr pellet method, Waltham, MA, USA) and X-ray photoelectron spectroscopy (XPS) (Thermo Scientific K-Alpha, Waltham, MA, USA). The crystalline phase was determined via X-Ray Diffraction (XRD) (PANalytical X’Pert PRO, Cu-Kα radiation, Malvern, UK). Optical performances were evaluated from UV-Vis absorption spectra (UV3600 spectrophotometer, Shimadzu, Kyoto, Japan), fluorescence emission (VARIAN spectrofluorometer, 25 °C, Palo Alto, CA, USA), and confocal fluorescence images (CLSM) (Olympus FV3000, 405 nm excitation, Olympus, Tokyo, Japan).

### 2.3. Preparation of BP-CDs

Fresh banana peel (30 g) was homogenized with 60 mL of deionized water using a high-speed mechanical homogenizer (Midea Group, Foshan, China) at room temperature to form a uniform slurry. This 1:2 (*w*/*v*) ratio was optimized for suitable viscosity. After adding 5 mL ethanol and thorough mixing, the mixture was subjected to hydrothermal treatment in a 100 mL Teflon-lined autoclave (Shanghai Yi Dian Physical Optical Instrument Co., Ltd., Shanghai, China) at 180 °C for 5 h. The naturally cooled product was centrifuged at 10,000 rpm for 10 min to remove large particulates. The supernatant was then dialyzed (MWCO: 500 Da) against deionized water for 8 h to obtain the purified BP-CDs solution, which was stored at 4 °C. The yield was approximately 10.5% based on the dry weight of the initial banana peel.

### 2.4. Detection Procedure for TC

Quantitative analysis of TC was performed using BP-CDs as fluorescent probes, based on the modulation of their fluorescence intensity. For detection, 3 mL of BP-CDs (0.005 mg/mL) solution was placed in a quartz cuvette, followed by the addition of TC solutions at varying concentrations (or other analytes). After homogenization for 1 min, the fluorescence spectra were recorded sequentially using an excitation wavelength of 360 nm.

### 2.5. Toxicity Assessment

An MTT assay was conducted to determine the cytotoxicity of BP-CDs on L929 and B16 cell lines. Cells were plated in 96-well plates (1 × 10^4^ cells/mL) (Cyagen Biosciences Inc., Suzhou, China) and cultured for 24 h. The culture medium was then exchanged for media supplemented with varying concentrations of BP-CDs (0–200 µg/mL). Following a 24 h exposure, the cells were rinsed with PBS and incubated with MTT solution (0.5 mg/mL) for 3 h. After removing the supernatant, the insoluble formazan was solubilized with DMSO, and the optical density at 490 nm was measured. Viability was calculated by normalizing the absorbance values to those of the control group.

### 2.6. Cellular Imaging and Intracellular Detection of Tetracycline

Following a 12 h culture in DMEM under standard conditions (37 °C, 5% CO_2_), adherent L929 cells were incubated for 1 h with BP-CDs (200 µg/mL) in fresh medium. After thorough PBS washes to eliminate unbound probes, cellular uptake was confirmed by CLSM. The capability for probing intracellular TC was assessed by treating the labeled cells with different TC concentrations for 5 min, after which the fluorescence quenching was immediately quantified via CLSM.

### 2.7. Data Processing

The experimental results are presented as mean values derived from triplicate measurements. XPS data were processed and analyzed with XPS Peak 4.1 software.

## 3. Results and Discussion

### 3.1. Morphology and Crystalline Properties of BP-CDs

**Figure 2:**
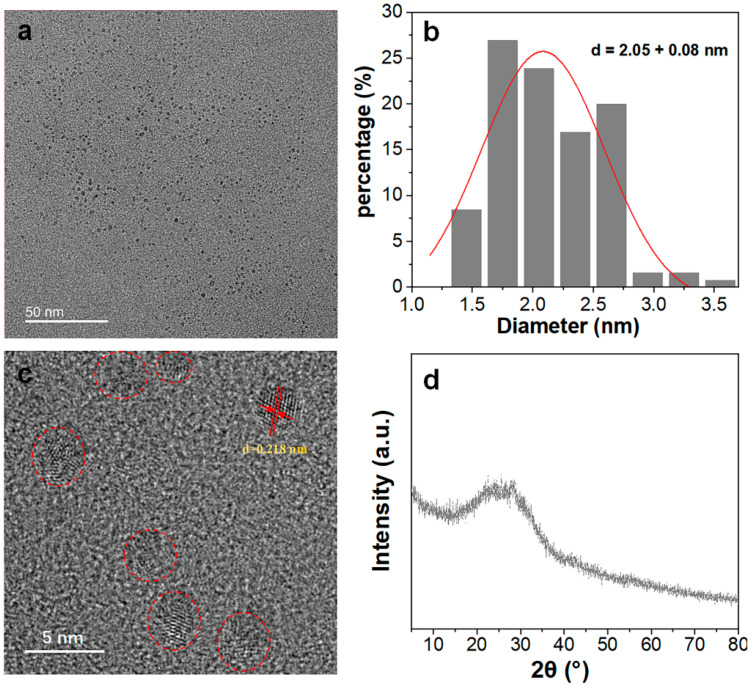
(**a**) TEM micrograph; (**b**) Particle size distribution histogram; (**c**) High-resolution TEM image and (**d**) XRD pattern of the synthesized BP-CDs.

BP-CDs with exceptional aqueous dispersibility were successfully synthesized from fresh banana peel via a one-step hydrothermal treatment at 180 °C for 5 h. This mild synthetic route, facilitated by a small amount of ethanol, required no additional chemicals, aligning with green chemistry principles. The relative photoluminescence quantum yield (QY) of BP-CDs synthesized over different durations (3–9 h) was evaluated using quinine sulfate as a standard (λ_ex_ = 360 nm). The QY was merely 0.6% after 3 h but stabilized between 2.0% and 2.2% for reaction times of 5–9 h, indicating optimal synthesis efficiency at 5 h, which was thus selected for subsequent studies. TEM imaging (Figure 2a) revealed that the BP-CDs are well-dispersed, spherical nanoparticles without significant aggregation. Particle size distribution analysis (Figure 2b) confirmed a narrow size distribution with an average diameter of 2.05 nm. High-resolution TEM (Figure 2c) displayed clear lattice fringes with a spacing of 0.218 nm, corresponding to the (100) lattice plane of graphitic carbon, indicative of localized nanocrystalline domains [26]. XRD analysis (Figure 2d) further supported this, showing a broad diffraction peak centered around 24°, characteristic of carbon-based materials with amorphous structure embedding small graphitic crystallites. The combined High-Resolution TEM and XRD results confirm that the BP-CDs comprise nanographitic domains within an amorphous carbon matrix.

### 3.2. Chemical Structure of BP-CDs

**Figure 3:**
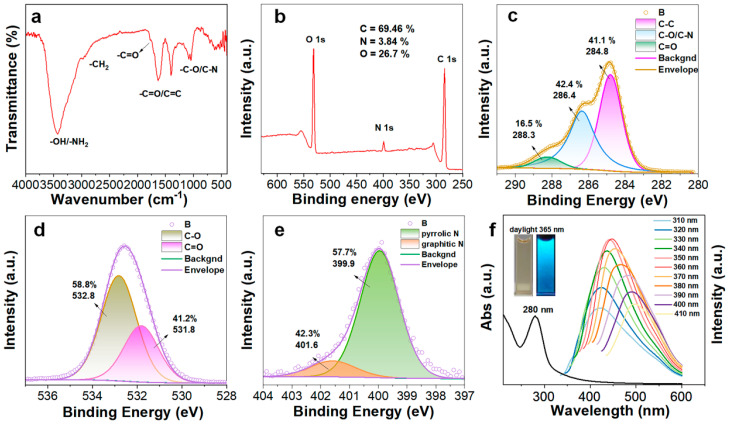
(**a**) FT-IR spectrum; (**b**) XPS survey spectrum; (**c**) High-resolution C 1s; (**d**) O 1s; (**e**) N 1s XPS spectra of BP-CDs and (**f**) UV-Vis absorption spectrum and fluorescence emission spectra under different excitation wavelengths (Inset: Photographs of BP-CDs solution under visible light (left) and 365 nm UV light (right)).

FT-IR spectroscopy was employed to characterize the surface functional groups of BP-CDs (Figure 3a). The spectrum exhibits a broad absorption band in the 3000–3500 cm^−1^ region, attributed to O–H and N–H stretching vibrations. The characteristic peaks at 2919 cm^−1^ and 2861 cm^−1^ correspond to aliphatic C–H stretching vibrations. An intense absorption band at 1635 cm^−1^ is characteristic of C=O stretching vibrations from aromatic C=C skeletal vibrations and/or carboxyl C=O stretching vibrations, confirming the presence of these oxygen-rich functional groups on the BP-CD surface. The absorption at 1398 cm^−1^ is associated with O–H bending vibrations, and the bands at 1089 cm^−1^ and 1047 cm^−1^ suggest the presence of C–O (alcohols, ethers) and/or C–N bonds.

Furthermore, XPS survey scan (Figure 3b) confirmed the elemental composition, revealing the presence of C (69.5%), O (26.7%), and N (3.8%). Deconvolution of the high-resolution C 1s spectrum (Figure 3c) yielded three characteristic peaks at 284.8 eV (C–C/C=C), 286.4 eV (C–O/C–N), and 288.3 eV (C=O from carboxyl groups), with relative concentrations of 41.1%, 42.4%, and 16.5%, respectively. The O 1s spectrum (Figure 3d) was fitted with two peaks at 531.8 eV (C=O in carboxyl) and 532.8 eV (C–O in hydroxyl/ether), accounting for 41.2% and 58.8%, respectively. The N 1s spectrum (Figure 3e) displayed characteristic peaks at 399.9 eV (pyrrolic N) and 401.7 eV (graphitic N). Collectively, the XPS results not only corroborate the FT-IR analysis, but also unequivocally confirm the co-existence of various functional groups, including carboxyl, amide, and hydroxyl groups, on the material surface. This unique surface chemistry, rich in oxygen-containing groups such as carboxyls, endows BP-CDs with excellent colloidal stability in aqueous media (maintaining stability for over two months) while enhancing interactions with target analytes, showing great potential for constructing high-performance fluorescent sensing platforms.

### 3.3. Optical Characterization of BP-CDs

**Figure 4:**
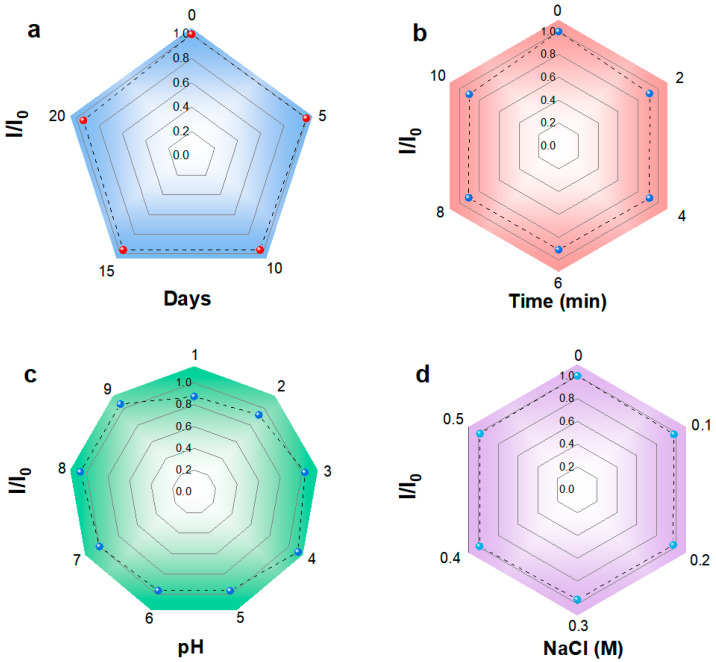
Stability of BP-CDs fluorescence intensity under different conditions: (**a**) Storage time; (**b**) Duration of UV irradiation; (**c**) pH and (**d**) NaCl concentration.

The optical characteristics of BP-CDs were thoroughly investigated. The UV-Vis absorption spectrum (Figure 3f) shows a prominent peak at 280 nm, ascribed to the π–π* transition of aromatic C=C bonds [27]. Fluorescence studies revealed excitation-dependent emission behavior, as the excitation wavelength increased from 310 nm to 410 nm, the emission maximum redshifted from 420 nm to 500 nm. The strongest fluorescence intensity was observed at λ_ex_/λ_em_ = 360/446 nm. This excitation-dependent emission is typical of carbon dots and is often attributed to surface state emissions [28]. Visually, the BP-CDs solution is light yellow and transparent under ambient light but emits intense blue fluorescence under 365 nm UV irradiation, consistent with the spectral data. The BP-CDs exhibited excellent stability under various environmental conditions. As shown in Figure 4a–d, the fluorescence intensity maintained 93.2% of its initial value after 20 days of storage, 90.3% after prolonged UV irradiation (10 min), over 87.5% across a wide pH range (1–9), and 98.5% under high ionic strength conditions (0.5 M NaCl). These stability percentages, marked directly on the corresponding spectra, demonstrate the robust chemical and photostability of BP-CDs, supporting their potential for sensing applications in complex environments.

This robust chemical and photostability underscores their suitability for sensing applications in complex environments. The BP-CDs possess a uniform nanosize, excellent dispersibility in water, a richly functionalized surface, strong and tunable fluorescence, and high stability, making them promising candidates for applications in fluorescent sensing, environmental monitoring, and bioimaging.

### 3.4. Detection of TC by BP-CDs

**Figure 5:**
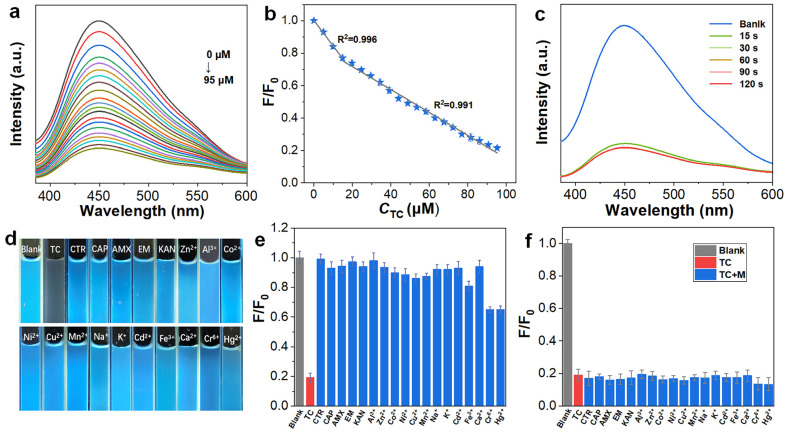
Evaluation of BP-CDs for TC sensing. (**a**) Fluorescence emission spectra upon addition of different TC concentrations (0–95 µM; λ_ex_ = 360 nm). (**b**) Corresponding linear calibration curves for fluorescence quenching (F_0_/F) versus TC concentration. (**c**) Fluorescence response kinetics upon addition of 100 µM TC. (**d**) Visual fluorescence under UV light before and after introducing different ions/antibiotics. (**e**) Selectivity profile showing F/F_0_ values in the presence of various substances (100 µM each). (**f**) Interference study comparing the response to TC alone and TC (100 µM) mixed with other substances (100 µM).

The widespread use of TC necessitates the development of rapid and sensitive detection methods to protect water resources and public health. In this study, synthesized BP-CDs were employed to construct a fluorescent sensing platform for TC detection. As shown in Figure 5a, the fluorescence intensity of BP-CDs at 445 nm exhibited a progressive decrease with increasing TC concentration (0–95 µM). Quantitative analysis (Figure 5b) established two linear calibration ranges: 0–15 µM (R2 = 0.996) and 15–95 µM (R2 = 0.992). The calculated limit of detection (LOD) was determined to be 0.191 µM (3σ/slope) [29], which surpasses most reported fluorescent probes for TC (Table 1), demonstrating exceptional detection sensitivity. The sensing platform exhibited rapid response characteristics, with the fluorescence signal stabilizing within 15 s after TC addition (Figure 5c), fully meeting the requirements for rapid detection. It is noteworthy that the sensing platform also exhibited significant fluorescence quenching towards other tetracycline antibiotics, namely oxytetracycline (OTC) and chlortetracycline (CTC), with well-defined linear concentration-response relationships (see Supplementary Figures S1–S4). This suggests that the BP-CDs probe possesses a certain degree of universality for detecting tetracycline antibiotics, which share a common core molecular structure.

To evaluate the selectivity and anti-interference capability of BP-CDs as a tetracycline sensing material, we systematically investigated their response characteristics toward common representative antibiotics—including penicillins (amoxicillin, AMX), cephalosporins (ceftriaxone sodium, CTR), chloramphenicols (chloramphenicol, CAP), aminoglycosides (kanamycin sulfate, KAN), and macrolides (erythromycin, EM)—as well as common metal ions such as K^+^, Na^+^, Ca^2+^, and Fe^3+^. The results demonstrate that BP-CDs exhibit highly specific selectivity toward tetracycline. As shown in Figure 5d, at the same concentration (100 µM), tetracycline induced particularly remarkable fluorescence quenching, while the quenching effects caused by other categories of antibiotics and common ions were negligible. The relative fluorescence intensity (F/F_0_) data (Figure 5e) quantitatively confirmed this specificity: the F/F0 value for tetracycline (19.1%) was significantly lower than that of other interfering substances. Except for Cr^6+^ and Hg^+^, which caused approximately 65% quenching, the quenching induced by other ions was almost negligible. Furthermore, anti-interference tests (Figure 5f) revealed that in the presence of tetracycline (100 µM), the additional fluorescence quenching contributed by these coexisting substances (100 µM) was negligible, confirming the reliability of the probe for application in complex matrices.

Therefore, the BP-CDs fluorescent probe combines advantages including a wide linear range, low detection limit, high selectivity, fast response, and strong anti-interference capability, making it a highly promising probe for practical TC detection applications. Notably, its effective response to other tetracyclines (OTC and CTC) further underscores its potential as a broad-spectrum sensing material for this antibiotic family.

### 3.5. Detection Mechanism of TC

**Figure 6:**
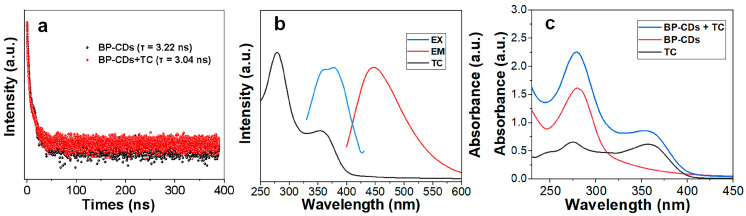
(**a**) Fluorescence decay profiles of BP-CDs before and after interaction with TC (100 µM); (**b**) Spectral overlap between the UV-Vis absorption spectrum of TC and the photoluminescence excitation and emission spectra of BP-CDs; (**c**) UV-Vis absorption spectra of TC, BP-CDs, and their mixture.

To elucidate the mechanism of fluorescence quenching of BP-CDs by tetracycline (TC), time-resolved fluorescence decay curves were analyzed. As shown in Figure 6a, the introduction of TC resulted in only a slight decrease in the average fluorescence lifetime of BP-CDs from 3.22 ns to 3.04 ns. Since Förster resonance energy transfer (FRET), as a typical dynamic quenching process, generally induces a significant reduction in fluorescence lifetime, the observed minimal change in lifetime falls far below the threshold required for FRET mechanisms. Therefore, FRET can be effectively ruled out as the dominant quenching pathway [36,37].

Subsequently, UV-Vis absorption spectroscopy was employed. Characterization of individual components (Figure 6b) revealed that TC exhibits a broad and intense absorption band in the 330–400 nm range, which exhibits a significant spectral overlap with the excitation spectra of BP-CDs. Critically, this absorption feature in the 330–400 nm range is unique to tetracycline antibiotics (TC, OTC, CTC), as other classes of tested antibiotics show no significant absorption in this region (see Supplementary Figure S5). This strong correlation between the presence of a characteristic absorption band and the observed quenching effect provides compelling evidence for the inner filter effect (IFE) as the dominant mechanism. In this process, TC molecules effectively compete for the absorption of excitation photons, thereby reducing the actual photon flux received by the BP-CDs [38].

To further corroborate this mechanism, the absorption spectrum of the BP-CDs/TC mixture was compared with the summed spectra of the individual components (Figure 6c). The experimental results indicate that the absorption spectrum of the mixture is merely a superposition, with no new absorption peaks observed. This key evidence excludes the possibility of ground-state complex formation [39]. As both static quenching (e.g., ground-state complex formation) and dynamic quenching (e.g., FRET) mechanisms are ruled out by the experimental data, we conclude that the inner filter effect is the dominant mechanism responsible for the fluorescence quenching of BP-CDs by TC.

Based on the confirmed IFE mechanism, we conducted an in-depth analysis of the bilinear relationship between the fluorescence signal (F/F_0_) and TC concentration observed in Figure 5b. This phenomenon has been reported in IFE-based sensing systems [40,41,42,43,44]. A reasonable explanation is that this bilinear relationship originates from the inherent concentration-dependent behavior of IFE: in the lower TC concentration range, the quenching efficiency exhibits an approximately linear relationship with the absorber concentration; when entering the higher concentration range, the significantly enhanced absorbance of TC reduces the effective excitation volume, causing the signal response to transition into a new linear regime with a different slope.

### 3.6. Cytotoxicity

**Figure 7:**
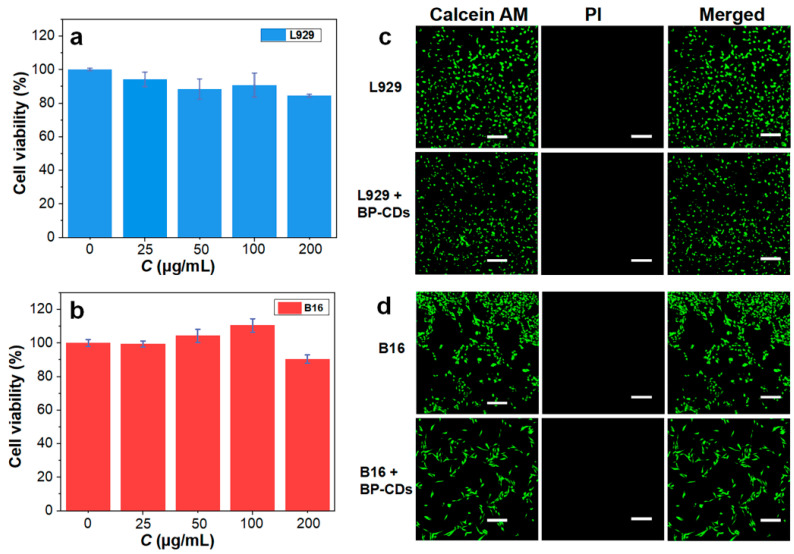
Cell viability of (**a**) L929 and (**b**) B16 cells after incubation with BP-CDs for 24 h. CLSM images of (**c**) L929 and (**d**) B16 cells, both with and without incubation of BP-CDs, which were co-stained using calcein AM (live cells, green) and PI (dead cells, red). The scale bar is 200 µm.

Excellent biocompatibility is an indispensable characteristic for biosensing probes. Therefore, we systematically evaluated the cytotoxicity of BP-CDs. MTT assay results demonstrated that both B16 cells and L929 cells exhibited remarkable biocompatibility with BP-CDs. After 24 h of incubation within the concentration range of 25–200 µg/mL, the cell viability rates of both cell types remained above 84%, with B16 cells maintaining viability consistently exceeding 90% (Figure 7a,b). To further verify these findings, live/dead cell double fluorescence staining was employed for morphological observation. Confocal laser scanning microscopy images revealed that both L929 and B16 cells treated with 200 µg/mL BP-CDs displayed intense green fluorescence from calcein-AM (indicating live cells), while no red fluorescence signal from propidium iodide (indicating dead cells) was observed (Figure 7c,d).

These results confirm an extremely low cell mortality rate, showing strong consistency with the MTT assay data. These findings demonstrate the low cytotoxicity of BP-CDs. Their excellent biocompatibility can be attributed to the following material characteristics: primarily, the abundant hydrophilic functional groups (such as hydroxyl and carboxyl groups) on the BP-CDs surface effectively reduce nonspecific interactions with cell membranes; additionally, the nanoscale carbon dot structure facilitates normal cellular internalization and metabolic processes, preventing intracellular accumulation that could lead to toxic effects. These characteristics together ensure the safety and reliability of BP-CDs for biosensing applications.

### 3.7. Intracellular Detection of TC

**Figure 8:**
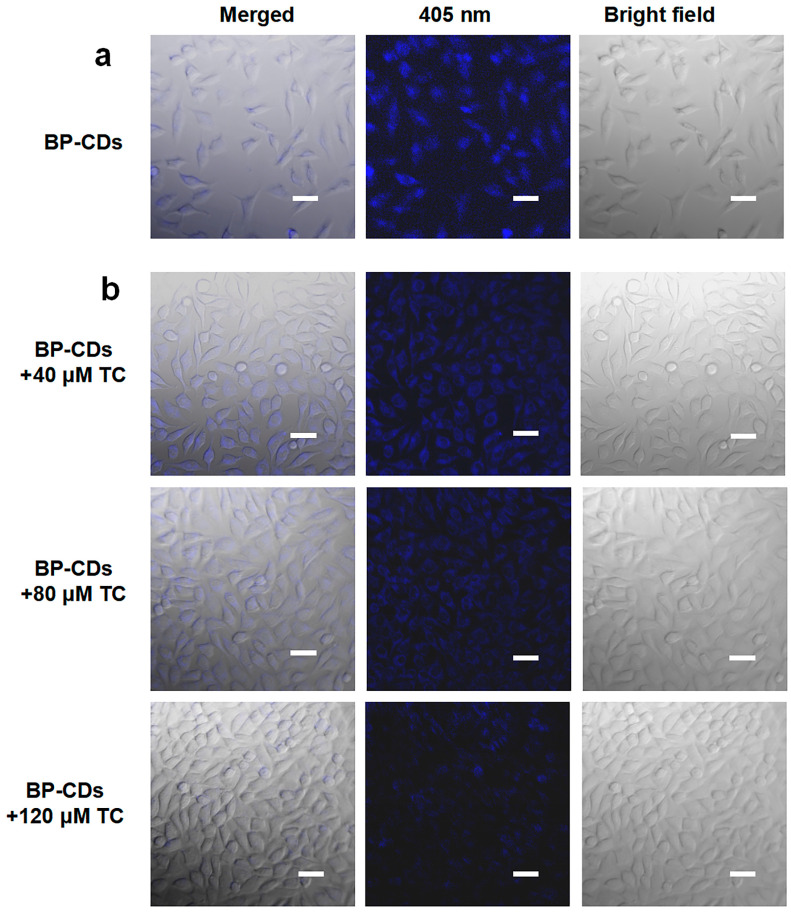
(**a**) CLSM images of L929 cells after incubation with BP-CDs (200 µg/mL); (**b**) CLSM images showing fluorescence quenching in BP-CD-labeled L929 cells upon treatment with different TC concentrations (40, 80, 120 µM). The scale bar is 50 µm.

Capitalizing on their low cytotoxicity and specific fluorescence response, the potential of BP-CDs for cellular imaging and intracellular TC monitoring was explored. CLSM observations revealed the promising potential of BP-CDs for cellular imaging (Figure 8a). After just 1 h of incubation at 200 µg/mL, intense blue fluorescence was observed within L929 cells, which can be attributed to efficient cellular internalization enabled by the material’s nanoscale dimensions and surface properties [45]. Furthermore, when L929 cells pre-labeled with BP-CDs were exposed to TC solutions at different concentrations (40, 80, and 120 µM), a clear concentration-dependent decrease in intracellular fluorescence intensity was observed (Figure 8b). This phenomenon demonstrates that BP-CDs can achieve semi-quantitative detection of intracellular TC through a fluorescence quenching mechanism, providing an effective means for real-time, in situ monitoring of the accumulation levels of the antibiotic TC within cells.

## 4. Conclusions

In this study, BP-CDs were successfully prepared from waste banana peels as a carbon source via a green hydrothermal method. The resulting material exhibits uniform particle size distribution (2.05 nm), good aqueous dispersibility, and stable blue fluorescence. Based on the IFE between BP-CDs and TC, a highly sensitive and rapid-response (within 15 s) fluorescent sensing platform was constructed, achieving a detection limit of 0.191 µM for TC. Cell experiments further confirmed that BP-CDs possess good biocompatibility (cell viability > 87%) and efficient cellular uptake capability, enabling real-time fluorescence imaging and semi-quantitative analysis of TC in living cells. This provides an effective tool for studying the uptake and distribution behavior of antibiotics at the cellular level.

Although this “waste-to-probe” strategy demonstrates promising application potential, challenges such as relatively low quantum yield and cross-response to other tetracycline antibiotics remain. Future research may focus on enhancing optical properties through doping or surface modification, expanding their application in the detection of multiple antibiotics, and promoting their validation in in vivo imaging and sensing. This study offers a new approach for the high-value utilization of agricultural waste and the development of green nanomaterials for environmental monitoring and biomedical applications.

## Figures and Tables

**Table 1:** Comparison of various fluorescent probes for TC detection.

Fluorescent Probe	Linear Range /μM	LOD /μM	Ref.
Co-MOF	0–250	4.71	[30]
Para-phenylenediamine-derived CDs	0.5–130	0.16	[26]
N-doped CDs	0–1	0.056	[31]
Cu-BL	0.5–80	0.27	[32]
OVA-AuNCs	1–30	0.563	[33]
boron nitride QD-g-C_3_N_4_BCN	1–400	0.29	[34]
Au NPs@MoS_2_	1–1100	0.41	[35]
BP-CDs	0–100	0.191	This work

## Data Availability

The original contributions presented in this study are included in the article/Supplementary Material. Further inquiries can be directed to the corresponding author.

## Supplementary Materials

The following supporting information can be downloaded at: https://www.mdpi.com/article/10.3390/ma18225211/s1, Figure S1: Evaluation of BP-CDs for CTC sensing; Figure S2: Fluorescence emission spectra upon addition of different CTC concentrations; Figure S3: Evaluation of BP-CDs for OTC sensing; Figure S4: Fluorescence emission spectra upon addition of different OTC concentrations; Figure S5: UV-Vis absorption spectra.
