# Supplementary material for: A Green-Synthesized Fluorescent Carbon Dot Probe Derived from Banana Peel for Cellular Imaging and Sensing of Tetracycline
Source: Materials (Basel). 2025 Nov 17;18(22):5211. doi: 10.3390/ma18225211 (PMC12654184; doi:10.3390/ma18225211)
Supplement: Supplementary file 1 [file materials-18-05211-s001.zip › materials-3967863-supplementary.pdf]

Supplementary Material

A Green-Synthesized Fluorescent Carbon Dot Probe Derived from Banana Peel for Cellular Imaging and Sensing of Tetracycline

Sihua Zeng [1,2,3], Chunrong Qin [3], Yuzhu Zhang [1,2], Haoyu Chen [3] and Hua Lin [1,2,*]

1 Guangxi Key Laboratory of Environmental Pollution Control Theory and Technology, Guilin University of Technology, Guilin, 541006, China; 202211002@hzxy.edu.cn (S.Z.); zhangyuzhu1128@163.com (Y.Z.)
2 Engineering Research Center of Watershed Protection and Green Development, Guilin University of Technology, Guilin, 541006, China
3 Guangxi Key Laboratory of Calcium Carbonate Resources Comprehensive Utilization, College of Materials and Chemical Engineering, Hezhou University, Hezhou, 542899, China; 14777562116@163.com (C.Q.); 19197800145@163.com (H.C.)
* Correspondence: linhua@glut.edu.cn

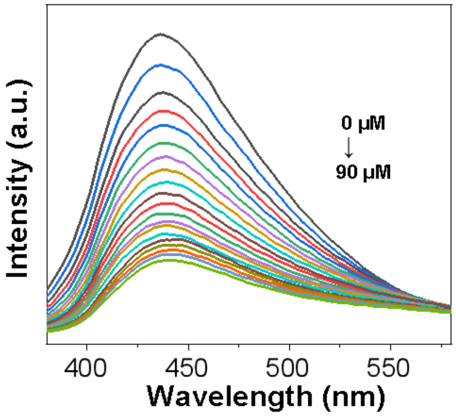

Figure S1. Evaluation of BP-CDs for CTC sensing.

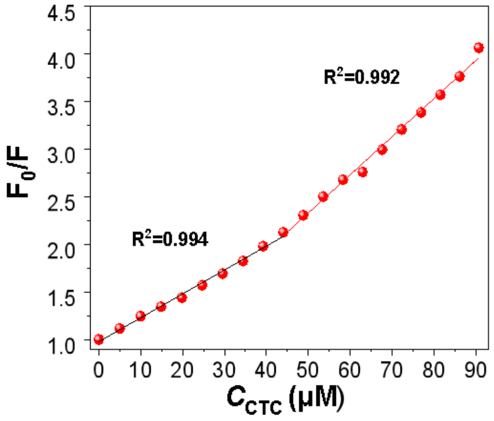

Figure S2. Fluorescence emission spectra upon addition of different CTC concentrations.

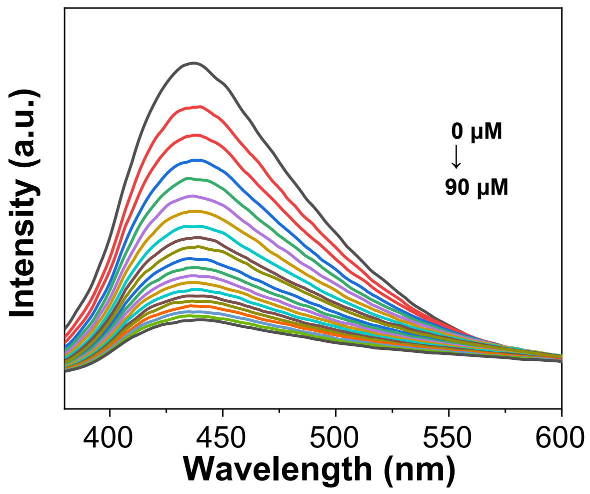

Figure S3. Evaluation of BP-CDs for OTC sensing.

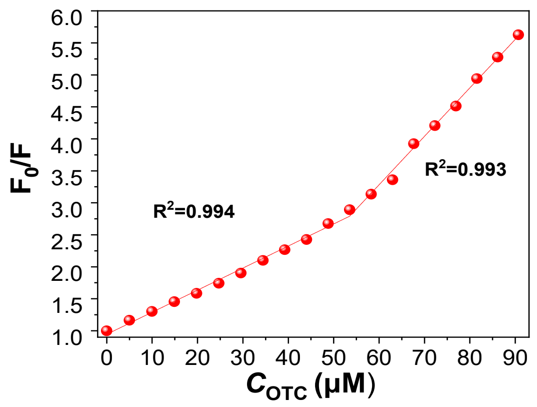

Figure S4. Fluorescence emission spectra upon addition of different OTC concentrations.

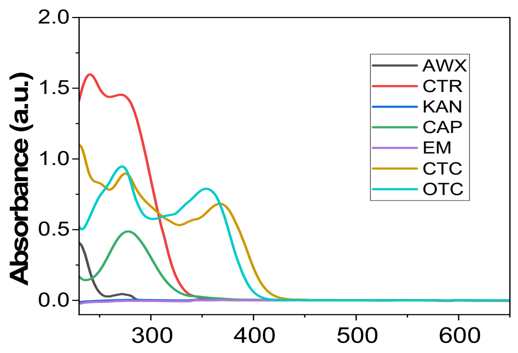

Figure S5. UV-Vis absorption spectra.
